# Identification of vancomycin-resistant enterococci clones and inter-hospital spread during an outbreak in Taiwan

**DOI:** 10.1186/1471-2334-13-163

**Published:** 2013-04-04

**Authors:** Sai-Cheong Lee, Mi-Si Wu, Hsiang-Ju Shih, Shu-Huan Huang, Meng-Jiun Chiou, Lai-Chu See, Liang-Kee Siu

**Affiliations:** 1 Division of Infectious Diseases, Chang Gung Memorial Hospital, Keelung, Chang Gung University, 222, Mai Chin Road, Kwei-Shan, Tao-Yuan, Taiwan; 2 Department of Nephrology, Chang Gung Memorial Hospital, Keelung, Chang Gung University, Kwei-Shan, Tao-Yuan, Taiwan; 3 Department of Laboratory Medicine, Chang Gung Memorial Hospital, Keelung, Taiwan; 4 Department of Public Health, Chang Gung University, Kwei-Shan, Tao-Yuan, Taiwan; 5 Biostatistics Core laboratory, Molecular Medicine Research Center, Chang Gung University, Kwei-Shan, Tao-Yuan, Taiwan; 6 Division of Clinical Research, National Health Research Institute, Miaoli, Taiwan

**Keywords:** VRE, MLST, Outbreak, Inter-hospital spread

## Abstract

**Background:**

In 2003, nosocomial infections caused by vancomycin-resistant enterococci (VRE) occurred rarely in Taiwan. Between 2003 and 2010, however, the average prevalence of vancomycin resistance among enterococci spp. increased from 2% to 16% in community hospitals and from 3% to 21% in medical centers of Taiwan. We used molecular methods to investigate the epidemiology of VRE in a tertiary teaching hospital in Taiwan.

**Methods:**

Between February 2009 and February 2011, rectal samples and infection site specimens were collected from all inpatients in the nephrology ward after patient consent was obtained. VRE strain types were determined by pulsed-field gel electrophoresis (PFGE) and multilocus sequence typing (MLST).

**Results:**

A total of 59 *vanA* gene-containing VRE isolates (1 per patient) were obtained; 24 originated from rectal sample surveillance of patients who exhibited no symptoms (22 *Enterococcus faecium* and 2 *Enterococcus faecalis*), and 35 had developed infections over 3 days after admission (32 *E. faecium,* 2 *E. faecalis*, and 1 *Enterococcus durans*). The 59 VRE isolates demonstrated vancomycin minimum inhibitory concentrations (MICs) of ≥256 μg/m. The MIC range for linezolid, tigecycline, and daptomycin was 0.25–1.5 μg/mL, 0.032–0.25 and 1–4 μg/mL, respectively. For 56 isolates, the MIC for teicoplanin was >8 μg/mL. The predominant types in the nephrology ward were MLST types 414, 78, and18 as well as PFGE types A, C, and D.

**Conclusion:**

VREs are endemic in nephrology wards. MLST 414 is the most predominant strain. The increase VRE prevalence is due to cross-transmission of VRE clones ST 414,78,18 by undetected VRE carriers. Because similar VRE STs had been reported in a different hospital of Taiwan, this finding may indicate inter-hospital VRE spread in Taiwan. Active surveillance and effective infection control policies are important controlling the spread of VRE in high risk hospital zones. All endemic VRE strains are resistant to teicoplanin but are sensitive to daptomycin, linezolid, and tigecycline.

## Background

For most immunocompetent patients, colonization with vancomycin-resistant enterococci (VRE) does not present a significant personal health risk; however, these patients may function as carriers, and following hospital admission, may pose a substantial risk for transmission [1-4]. In 2003, nosocomial infections caused by VRE occurred rarely in Taiwan [5]. Between 2003 and 2010, however, the prevalence of vancomycin resistance among enterococci spp. in community-hospitals and medical centers has increased from 2% to 16% and from 3% to 21%, respectively [5]. Little is known about the epidemiology of VRE, and most information has derived from the descriptions of monoclonal outbreaks [6-11]. The reasons underlying the rapid emergence of VRE had not been investigated in Taiwan thus far. Taiwan’s current management guidelines for VRE colonization and infection mimic those of the United States, which involve reasonably strict isolation measures [3,6]. The complete enforcement of these policies for VRE-colonized patients is difficult and impractical; the isolation rooms in most teaching hospitals are inadequate and high in cost [7]. Thus, we used multi-locus sequence typing (MLST) and pulsed-field gel electrophoresis (PFGE) to assess the epidemiology of VRE in a hospital setting, investigate the need for these policies, and discover new VRE clones. We also investigated the in-vitro susceptibilities of VRE to current antimicrobial agents.

## Methods

### Setting and study design

Chang Gung Memorial Hospital at Keelung in Taiwan is a 1088-bed, tertiary-care, teaching hospital. The prevalence of vancomycin resistance among enterococci spp. in this hospital rose from 10% in 2003 to 30% in 2009, and it was most pronounced in the nephrology ward. A VRE outbreak was suspected in the nephrology ward because the prevalence rate, 30%, was higher than the average rate,16%, in Taiwan [5]. This research plan was approved by the Human Trial and Ethics Committee of Chang Gung Memorial Hospital on December 24, 2008 (reference number 97-2117B). Between February 2010 and February 2011, a VRE surveillance study was conducted on both hemodialysis and non-hemodialysis inpatients in the nephrology ward of this hospital. Rectal swab cultures for VRE were collected from all nephrology inpatients during admission after patient consent was obtained. Colonization was defined as VRE isolation from rectal swabs in the absence of infection symptoms or signs. Infection was defined as VRE isolation from a sterile or non-sterile site along with the presentation of fever, leukocytosis, and other signs caused by the VRE.

All VRE-infected patients were confined to a single room or a double room with two beds for 2 VRE-infected patients of the same sex. All health-care workers (HCW) who administered care on VRE-infected patients were asked to follow infection control policies during patient care, including hand washing and glove and gown wearing when necessary [12]. During this study period, active surveillance was performed on inpatients of the nephrology ward after patient consent was obtained. Further, within this study period, potential VRE specimens were collected both from HCWs after they provided consent and from the nephrology ward environment, including bedrails, pillows, tables, door handles, blood-pressure cuffs, ventilator monitor surfaces, and the surfaces of medical devices such as EKG monitors. All VRE strains isolated from inpatients, HCWs, and the environment were stored until needed for epidemiological and antibiotic susceptibility studies.

### Identification of VRE

All rectal swabs were cultured on blood agar plates, which were examined after 48 h of incubation at 37°C. The colonies were identified as those of *Enterococcus* spp. based on known enterococcus characteristics, including the presence of gram-positive cocci, optochin resistance, bile-esculin color change to black, and growth in 6.5% sodium chloride (NaCl) [1,2]. Specific enterococcus spp. were identified by differential utilization of arginine, sorbitol, arabinose, and raffinose and by the rapid 32 Strep kit test (bioMerieux Vitek Inc., Hazelwood, Missouri, USA) [1,2]. VRE presence was confirmed by growth in brain heart infusion agar that contained 6 μg/mL vancomycin [13,14].

### Pulsed-field gel electrophoresis

For PFGE analysis, the isolates were inoculated into 5-mL nutrient broth and incubated for 3 h at 37°C with shaking to achieve exponential growth. Agarose plugs were prepared from the cultures, and within the plugs, the bacterial cells were lysed by proteinase K. Extracted genomic DNA was digested with the restriction endonuclease *Sma*I [15,16]. The resulting restriction fragments were separated by PFGE using the MAPPER system (Bio-Rad Laboratory, Hercules, CA, USA). Band patterns were analyzed to determine clonal identity. Previously described criteria were used for the analysis of genomic DNA [15,16].

### Multilocus sequence typing

VRE isolates were typed by MLST. With the use of the Ibis T5000™ Biosensor System (Abbott, USA), we amplified 7 selected gene fragments that encode 7 housekeeping proteins by broad-range polymerase chain reaction (PCR). The base compositions of the amplicons were determined by electrospray ionization mass spectrometry. The base compositions of different target regions are shown by mass spectrometry and were used to create a signature that distinguished strains from one another [17].

### Genotypic analysis of resistance pattern of VRE

To identify possible additional epidemiological markers, we investigated the presence of *van*A, *van*B, *van*C1, and *van*C2 genes by PCR. The PCR primer sequences were based on the published genes for *Enterococcus faecalis*, *Enterococcus faecium*, and *Enterococcus gallinarum*[18,19].

### Antibiotic susceptibility

The VRE isolate MICs for 8 antimicrobial agents, including daptomycin (Cubist Pharmaceuticals), fusidic acid (Leo), linezolid (Pfizer), mupirocin (GlaxoSmithKline), teicoplanin (Sanofi-Aventis), tigecycline (Pfizer), trimethoprim/sulfamethoxazole (Sandoz), and vancomycin (Eli Lilly), were determined by Etest (AB Biodisk, Solna, Sweden) according to the published guidelines [13,14]. The MIC ranges for these antibiotics were as follows: daptomycin, 0.002–32 μg/mL; fusidic acid, 0.016–256 μg/mL; linezolid, 0.016–256 μg/mL; mupirocin, 0.064–1024 μg/mL; teicoplanin, 0.016–256 μg/mL; tigecycline, 0.016–256 μg/mL; trimethoprim/sulfamethoxazole, 0.002–32 μg/mL; and vancomycin, 0.016–256 μg/mL. As a control strain, *E. faecalis* ATCC 29212 was included with acceptable MIC limits according to CLSI M100-S19 (January 2009): daptomycin, 1–4 μg/mL; linezolid, 1–4 μg/mL; teicoplanin, 0.125–0.5 μg/mL; tigecycline, 0.03–0.12 μg/mL; and vancomycin, 1–4 μg/mL.

## Results and discussion

A total of 59 VRE isolates were obtained from 59 patients (Table 1). For surveillance purposes, 101 rectal swabs were collected and cultured from 101 inpatients after admission; 24 of these inpatients were culture-positive for VRE. These 24 VRE isolates were indicative of colonization without any clinical symptoms or signs of VRE infection and all carried the *van*A gene (22 *E. faecium* and *2 E. faecalis* isolates). The remaining 35 isolates were discovered from 35 in patients with clinically manifested infections caused by VRE. All these 35 infections caused by VRE developed over 3 days after admission and were considered as healthcare-associated infections. These 35 isolates of VRE causing infections also carried the *van*A gene and consisted of 32 *E. faecium, 2 E. faecalis* and 1 *E. durans*. Of the 54 *E. faecium* isolates, 35 were type ST 414, 9 were ST 18, 4 were ST 78, 3 were ST 203, 2 were ST 341, and 1 was ST 556 (Table 1). Of the 35 ST 414 *E. faecium* isolates, 17 were PFGE subtype A3, 8 were subtype A4, 5 were subtype A1, 3 were subtype A5, 1 was subtype A2, and 1 was subtype A6 (Table 1). Of the 9 ST 18 *E. faecium* isolates, 5 were PFGE subtype D1, 2 were subtype D2, and 2 were subtype D3 (Table 1). The PFGE types of remaining ST types of *E. faecium* isolates belonged to types B through F: 3 B1, 1 C1, 2 C2, 1 C3, 2 E1, and 1 F1 (Table 1). Of the 4 *E. faecalis* isolates, 3 were ST 414 and 1 was ST 203. The PFGE subtypes of these 3 ST 414 *E. faecalis* isolates were identical, G1 (Table 1). The single *E. durans* isolate belonged to ST 341. The 24 colonized VRE isolates and the 35 infection isolates all were discovered in the same nephrology ward that was under the care of the same medical team, including attending physicians, resident doctors, and nurses within the study period, February 2010 through February 2011. These pieces of epidemiologic evidence strongly support that cross-transmission had occurred in the nephrology ward.

**Table 1:** Molecular types, antibiotic susceptibility and basic data of 59 VRE isolates from nephrology inpatients

**Inf/Col(N)**	***van*A gene**	**MLST type(N)**	**PFGE (N)**	**Site of specimen(N)**	**MIC ug/ml mean/range**							
					**Van**	**Teic**	**Fusi**	**Mup**	**Line**	*TS	**Tige**	**Dapt**
Inf (32) (*E.faecium*)	(+)	414(19)	A3(8)	B(2),P(3) U(3)	>256	157.8/6- > 256	2.18/1.5-3	0.50/0.38-1	0.99/0.38-1.5	>32	0.07/0.047-0.125	1.66/0.8-2
			A4(6)	B(1), C(1), P(1), U(2), W(1),	>256	192.67/4- > 256	2.17/1.5-3	0.42/0.38-0.5	0.73/0.38-1	>32	0.067/0.047-0.094	2.42/1.5-3
			A5(3)	U(2),W(1)	>256	>256	3/3-3	0.42/0.38-0.5	1.17/1-1.5	>32	0.064/0.064-0.064	2.17/1.5-3
			A1(2)	W(2)	>256	>256	2.5/2-3	0.38/0.25-0.5	0.88/0.75-1	>32	0.064/0.064-0.064	2/2-2
		18(8)	D1(4)	B(1), P(1), U(1) W(1),	>256	148/32- > 256	1.88/1.5-2	0.38 0.25-0.5	0.66/0.38-1	16.04/0.032- > 32	0.17/0.094-0.38	2.25/2-3
			D2(2)	U(2)	>256	192/128- > 256	2/2-2	0.25/0.25-0.25	1/0.5-1.5	16.02/0.032- > 32	0.11/0.094-0.125	3.5/1-6
			D3(2)	B(1), W(1)	>256	72/48-96	2.5/2-3	0.38/0.38-0.38	0.75/0.5-1	16.13/0.25- > 32	0.11/0.094-0.125	2.75/1.5-4
		78(3)	C1(1)	U(1)	>256	32	3	0.38	0.25	>32	0.125	3
			C2(1)	U(1)	>256	64	1.5	0.25	0.38	>32	0.064	3
			C3(1)	W(1)	>256	16	1.5	0.25	0.38	>32	0.064	4
		341(1)	E1(1)	U(1)	*>256*	>256	2.0	0.5	1.5	>32	0.094	2
		556(1)	F1(1)	U(1)	>256	128	3	0.38	1.5	>32	0.064	2
Col (22) (*E.faecium*)	(+)	414(16)	A3(9)	RS(9)	>256	194.7/24-256	2.72/1.5-4	2.11/0.25-16	0.83/0.25-1.5	>32	0.08/0.047-0.19	2.39/1.5-4
			A1(3)	RS(3)	>256	213.33/128-256	3/3-3	0.46/0.38-0.5	1.08/0.75-1.5	>32	0.07/0.047-0.094	2.33/2-3
			A4(2)	RS(2)	>256	>256	2.25/1.5-3	0.5/0.5-0.5	0.63/0.25-1	>32	0.055/0.047-0.064	3/2-4
			A2(1)	RS(1)	>256	24	3	0.38	1	>32	0.047	3
			A6(1)	RS(1)	>256	>256	2	0.5	1.5	>32	0.25	1.5
		203(3)	B1(3)	RS(3)	>256	85.3/64-128	1.71/0.1-3	0.42/0.38-0.5	1.06/0.5-1.5	>32	0.146/0.125-0.19	3.17/1.5-4
		18(1)	D1(1)	RS(1)	>256	32	4	0.5	0.5	>32	0.125	1
		78(1)	C2(1)	RS(1)	>256	128	2	0.38	1	>32	0.064	2
		341(1)	E1(1)	RS(1)	>256	>256	3	0.38	1	>32	0.047	2
Inf (2) (*E.faecalis*)	(+)	414(2)	G1(2)	U(1), W(1)	>256	18/12-24	2/2-2	0.38/0.38-0.38	1.25/1.0-1.5	>32	0.055/0.047-0.064	1.75/1.5-2
Col (2) (*E.faecalis*)	(+)	414(1)	G1 (1)	RS(1)	>256	>256	2	0.5	1.5	>32	0.094	4
		203(1)	H1(1)	RS(1)	>256	128	2	0.5	1.5	>32	0.19	4
Inf (1) (*E.durans*)	(+)	341(1)	I1(1)	U(1)	>256	96	3	0.5	1	>32	0.047	1.5

*Inf*, infection; *Col*, colonization; *MLST*, multilocus sequence typing; *PFGE*, pulsed-field gel electrophoresis.

*B*, blood, *C*, catheter tip, *P*, pus, *RS*, rectal swab, *U*, urine, *W*, wound.

*MIC*, minimal inhibitory concentrations; *Van*, vancomycin; *Tei*, teicoplanin; *Fusi*, fusidic acid; *Mup*, mupirocin; *Line*, linezolid; *TS*, *trimethoprim/sulfamethoxazole (1/19); *Tige*, tigecycline; *Dapt*, daptomycin.

Of the 227 samples potentially containing VRE that were collected from the environment and medical devices, only 1 VRE isolate was discovered on a patient pillow. The room of this patient had hosted a VRE-infected patient within the same week. This environmental VRE isolate was identified as *E. faecium* MLST 414 and PFGE type A3. Only 23 nurses and 2 resident doctors consented to hand culture for bacteria, and all cultures were negative. ST types 414, 18, and 78 were isolated from both colonized and infected patients (Table 1). Epidemiologic links evident among similar VRE ST types of colonized and infected patients and the environment indicate that this VRE outbreak most likely was due to cross transmission from the inpatients and the environment—probably originating from undetected VRE carriers.

Of the 59 VRE isolates, 56 demonstrated teicoplanin MIC of >8 μg/mL (Table 1). The MIC range was 0.25–1.5 μg/mL for linezolid, 0.032–0.25 μg/mL for tigecycline, and 1–4 μg/mL for daptomycin.

This study revealed a close relationship between VRE colonization and VRE symptomatic infections; similar ST types (414, 78, 18, and 341) and PFGE types (A, C, D, and E) were identified in patients both asymptomatic for and clinically manifested VRE. This finding indicates that infection control policies for VRE will not be successful if the policy includes clinically manifested VRE infections and excludes asymptomatic VRE colonization. If the infection control policy also includes asymptomatic VRE colonization, then active VRE surveillance will be required. Further study is needed to evaluate the timing and conditions under which active VRE surveillance should be initiated and can be proven as cost-effective. Although we had discovered only 1 VRE isolate from the environment, other possible environmental sites for contamination still exist; chairs and couches can become contaminated via perianal contact [7]. For HCWs administering care to VRE patients, the most common sites of contamination were gowns and gloves according to a prior report [7]. Because only 1 VRE isolate was discovered from the environment in this study, cross transmission of VRE in the nephrology ward may occur via HCWs, including resident doctors and attending physician, who refused the hand culture for bacteria. Although we were unable to isolate VRE from the 23 nurses and 2 doctors who had consented to hand culture, the patients’ hands apparently are a frequent site of contamination [20,21]. Routine patient use of alcohol-chlorhexidine hand gel or appropriate hand-washing practices upon entry to and departure from the hemodialysis area and nephrology ward should be encouraged [3,6]. The links between VRE acquisition and the hospital environment is recognized in current patient care guidelines, which seek to limit VRE transmission [1,6].

Cho *et al.* in Korea reported VRE ST 192, ST 78, ST 17, and ST 414 (highest to lowest frequency) in 2011 [22]. In our study, however, VRE ST 414 was the most frequent type. In Taiwan, Lu *et al.* performed MLST on 149 VRE blood isolates obtained between 2003 and 2010 [23]. Between 2009 and 2010, ST 18 and ST 414 were the 2 predominant STs, and accounted for 29.7% and 25.0% of all isolates, respectively [23]; however, the epidemiological relationships between these ST types were not reported. In our study, we reported an outbreak of VRE colonization and infection caused by ST 414 and 18. The identification of similar VRE STs in different hospitals of the same country may indicate inter-hospital VRE spread in Taiwan. Similar VRE STs were detected in Korean and Taiwanese hospital, which indicates that international spread of VRE is possible. An effective infection control policy is needed to prevent inter-hospital and international VRE spread.

## Conclusions

In Taiwan, increased VRE prevalence is due to cross transmission of VRE clones ST 414, 78, and 18 from undetected VRE carriers. To avoid cross transmission of VRE in hospital wards, an infection control policy for VRE should include asymptomatic VRE colonization, and thus, active surveillance of VRE during admission; subsequent isolation and appropriate hand-washing practices may be necessary to prevent the spread of VRE within a hospital. Because similar VRE STs had been reported in a different hospital of Taiwan, inter-hospital VRE spread may exist in Taiwan. Because of the high likelihood of environmental contamination by VRE-colonized or VRE-infected inpatients, we believe it is imperative that the ward environments are cleaned thoroughly on a daily basis throughout a VRE patient’s hospitalization and also after discharge.

## Abbreviations

VRE: Vancomycin-resistant enterococcus; Col: Colonization; Inf: Infection; Van: Vancomycin; Tei: Teicoplanin; Fusi: Fusidic acid; Mup: Mupirocin; Line: Linezolid; TS: Trimethoprim/sulfamethoxazole; Tige: Tigecycline; Dapt: Daptomycin; MLST: Multilocus sequence typing; PFGE: Pulsed-field gel electrophoresis; B: Blood; C: Catheter tip; P: Pus; RS: Rectal swab; S: Stool; U: Urine; W: Wound.

## Competing interests

The authors declare that they have no competing interests.

## Authors’ contributions

SCL conducted the molecular genetic studies, participated in sequence alignment, and drafted the manuscript. MSW conceived the study and participated in its design and coordination. SHH isolated the VRE and performed antimicrobial susceptibility testing. HJS performed the *van* resistance gene typing experiments, PFGE, and MLST. MJC and LCS conducted statistical analysis. LKS assisted with PFGE and MLST. All authors read and approved the final manuscript.

## Pre-publication history

The pre-publication history for this paper can be accessed here:

http://www.biomedcentral.com/1471-2334/13/163/prepub
